# Perceptions of Online Learning Implementation in Dental Education during the COVID-19 Pandemic: A Cross-Sectional Study of Dental School Faculty Members in Southeast Asia

**DOI:** 10.3390/dj11090201

**Published:** 2023-08-23

**Authors:** Yuniardini Septorini Wimardhani, Ratna Kumala Indrastiti, Adrianti Primata Ayu, Anandina Irmagita Soegyanto, Indriasti Indah Wardhany, Ajiravudh Subarnbhesaj, Nik Mohd Mazuan Nik Mohd Rosdy, Thao Thi Do

**Affiliations:** 1 Department of Oral Medicine, Faculty of Dentistry, Universitas Indonesia, Jl. Salemba Raya No.4, Central Jakarta 10430, Indonesia; ratna.kumala02@ui.ac.id (R.K.I.); anandina.irmagita74@ui.ac.id (A.I.S.); indriasti.indah@ui.ac.id (I.I.W.); 2 Dentistry Program, Faculty of Dentistry, Universitas Indonesia, Jl. Salemba Raya No.4, Central Jakarta 10430, Indonesia; adrianti.primata@ui.ac.id; 3 Oral Epidemiology and Clinical Studies Research Cluster, Faculty of Dentistry, Universitas Indonesia, Jl. Salemba Raya No.4, Central Jakarta 10430, Indonesia; 4 Department of Oral Biomedical Sciences, Division of Oral Diagnosis, Faculty of Dentistry, Khon Kaen University, 123 Mittraparp Rd., Muang, Khon Kaen 40002, Thailand; ajirsu@kku.ac.th; 5 Centre of Oral & Maxillofacial Diagnostics & Medicine Studies, Faculty of Dentistry, Universiti Teknologi MARA, Sungai Buloh Campus, Jalan Hospital, Sungai Buloh 47000, Selangor, Malaysia; nikmohd@uitm.edu.my; 6 Department of Oral Pathology and Periodontology, Faculty of Odonto-Stomatology, Can Tho University of Medicine and Pharmacy, 179 Nguyen Van Cu Street, An Khanh, Ninh Kieu, Can Tho, Vietnam; dtthao@ctump.edu.vn

**Keywords:** dental education, perception, COVID-19 pandemic, hybrid learning, online learning

## Abstract

Objective: To assess the perceptions of faculty members from dental schools in Southeast Asian countries regarding the implementation of online learning during the COVID-19 pandemic. Methods: A previously implemented questionnaire comprising 43 questions was utilized in this study. Lecturers from four universities in Southeast Asia were invited to participate in the study. Statistical analysis: The data were analyzed using SPSS version 25.0 through several types of comparative and correlation analyses. Results: There were 183 lecturers who participated in the study. The overall responses suggest that the perceived effectiveness of online learning in dentistry was centered on a neutral value. The participants faced challenges when implementing online learning during the COVID-19 pandemic, with the lack of interaction being the most challenging factor. They agreed that online learning had many advantages, specifically in time flexibility and communication. The participants had stronger perceptions relating the advantages and opportunities of online teaching, and recognized that the effectiveness of offline teaching alone was limited. Conclusion: The perceptions of Southeast Asian dental school faculty members were inclined toward a positive outlook on blended learning for implementation in dentistry, as a means of providing opportunities to use online learning beyond COVID-19 in the future.

## 1. Introduction

The COVID-19 pandemic has significantly impacted dental education worldwide. The implementation of distance learning in the context of dental education has created challenges for many dental schools in meeting student competence and training needs. The challenges are rooted to the nature of the dental curriculum, which consists of both theoretical and intense practical training. Dental education combines didactic, laboratory, and clinical psychomotor skill development. The program produces graduates who are clinically competent and independent. Throughout this educational journey, students acquire psychomotor skills while upholding an appropriate level of personal values (including professionalism), as well as the underlying theories and concepts of relevant disciplines. The improvement and development of problem-solving skills, critical thinking, reflective practice, and the capacity to forge dynamic relationships with patients and teams are further components of the journey [1]. Previous studies addressing dental education challenges during the COVID-19 period included infrastructure, access to pre-clinical and clinical training, the mode of examinations, and graduation requirements [2,3,4,5].

Many studies from different parts of the world have evaluated students’ attitudes toward, perceptions of, and impact of online education in dentistry during the COVID-19 pandemic [6,7,8,9]. Although dental students adapted to online learning, they agreed that blended learning could be implemented using innovative education technologies that also consider the education pedagogy [10,11,12]. However, limited studies have explored the perceptions of staff, faculty, and administrators during COVID-19 [9,13].

A report from a single-institution study in Denmark during the pandemic showed that both students and faculty members had positive perceptions of online learning. The study was conducted at the beginning of the pandemic [13]. A qualitative study in a Singaporean dental school highlighted the challenges with online learning; however, the academicians were confident about its future in dentistry education [14]. Previous studies have explored the perceptions of social science faculty members and students in Jordan regarding online learning during the COVID-19 pandemic [15]. This study showed that more effort was needed to achieve interactive learning, and the lack of interaction resulted in low student performance, although lecturers agreed that the method of learning could differentiate student performance and help them achieve academic competence. However, whether these results can be replicated in dentistry education is still unconfirmed [13]. Previous studies on dental hygienist educators have explored the level of personal burnout that may influence teaching efficacy [16].

A study on the effects of online learning for dental students within different dental schools in seven Asian countries and other parts of the world have demonstrated that blended learning provides better learning efficiency [12,13,14]. The study participants comprised dental students from different regions of Asia, hence they may have had different conditions during the COVID-19 pandemic. The study also had no data on the pandemic’s effect on lecturers. Furthermore, the study contained limited data relating tor dental schools in the Southeast Asian region. Therefore, the aim of this study was to assess the perceptions of faculty members from different dental schools in Southeast Asian countries regarding the implementation of online learning during the COVID-19 pandemic.

## 2. Methods

The present study was first approved by the Research Ethics Committee of the Faculty of Dentistry Indonesia (no. 62/Ethical Approval/FKGUI/IX/2022) and was also reviewed and approved by each dental school’s ethics committee. All participants of the research agreed and provided informed consent.

### 2.1. Questionnaire

The questionnaire employed in this study was previously used in a study by Almahasees in 2021 [15]. Each item of the questionnaire has been reviewed by experts for relevance and clarity; therefore, it was relevant to the conceptual framework of online learning. There were 43 questions divided into 5 sections (demographic characteristics, information technology skill and preparation, effectiveness, challenges, and advantages of online learning during the COVID-19 pandemic). Each school’s primary investigator was responsible for performing the translation and back-translation of the questions into participants’ native languages, to ensure that the questions were understood.

### 2.2. Study Participants

All lecturers from each dental school (Universitas Indonesia, Universiti Teknologi MARA, Khon Kaen University, and Can Tho University Medicine and Pharmacy) were invited to participate in the study. An invitation was sent by each school’s main investigator using the WhatsApp messaging application (Mountain View, CA, USA). Invitations were also sent to all lecturers from each school; however, only lecturers who taught at the dental school for more than one year were included in the study.

### 2.3. Statistical Analysis

The data generated from the study were analyzed using SPSS version 25.0(SPSS Inc., Armonk, NY, USA). Several comparative analyses (continuity correction, Pearson’s chi-square, Mann–Whitney, Kruskal–Wallis, and a one-way analysis of variance) and correlation analysis (Kendall and Spearman) were used.

## 3. Results

### 3.1. Participant Characteristics

There were 183 lecturers from four dental schools who agreed to participate in the study. Seven participants with less than one year of teaching experience were excluded from the study, leaving 176 participants who met the inclusion criteria, and the response rate was 57%. Table 1 illustrates participant characteristics.

Most participants were responsible for teaching activities in pre-clinical and clinical programs for a dental course. Almost half of the participants had experience teaching online before the COVID-19 pandemic, and half had also received training about online teaching from their universities before and during the pandemic. However, less than 30% received training for online teaching from external institutions before and during the pandemic. The most common platform for online teaching used by most participants from all dental schools was Zoom, and WhatsApp and the chat feature provided by the learning platform were used to communicate with the students.

### 3.2. Information Technology Skills and Preparation for Online Learning

Table 2 illustrates the results of the second part of the questionnaire, which was used to explore participants’ computer literacy and preparation for online teaching. Most respondents agreed that they had enough IT skills required for online learning. Very few participants thought that they did not have the necessary IT competency to teach online. The participants also agreed that more effort in class preparation was needed when performing online teaching. Although the participants agreed that online platforms had the required tools to facilitate online classes, they believed that utilizing the camera maximized class interactions and effectiveness.

### 3.3. Faculty Perceptions of the Effectiveness of Online Learning

Table 3 shows the results of the third part of the questionnaire on respondents’ attitudes toward online learning effectiveness. The responses were neutral on the first, seventh, and ninth items. The respondents disagreed with the statement that practical classes could be taught without real interaction between the lecturers and the students. The respondents also disagreed with the statement that students studying using online learning will perform better than students studying face-to-face. On the other hand, respondents agreed on the remaining items in the section. The overall responses for the perceptions of the effectiveness of online learning in dentistry were centered on a neutral value (3.4 ± 0.43).

### 3.4. Challenges of Online Learning

Overall, the study participants agreed that they faced challenges during the employment of online learning during the COVID-19 pandemic (3.7 ± 0.57). A lack of interactions was the factor with the highest score (4.1 ± 0.84), followed by internet issues, technical issues, adjustments for students with hearing disabilities, time management, and data privacy and security (Table 4).

### 3.5. Perceptions of the Advantages of Online Learning

The faculty members participating in the study agreed that online learning had many advantages (the overall score was 4.0 ± 0.6). They agreed that online learning’s greatest advantage was time flexibility and communication (4.2 ± 0.75). The study participants also concurred that online learning provided comfort, low cost, and the opportunity to use new tools and grasp new skills (Table 5).

### 3.6. Correlation between Participant Characteristics and Their Perceptions of Online Learning

Correlation analyses between participant characteristics and their perceptions of online teaching were performed (Table 6). The study analysis revealed that a significant negative correlation existed between the perception of IT skills with age (r = −0.207) and teaching experience (r = −0.203). The results showed that older participants with a longer teaching experience had lower perceptions about their IT skills in online teaching. The correlation analyses were not significant for the other variables tested.

### 3.7. Overall Perceptions of Online Learning

After answering the previous sections of the questionnaire, the respondents were finally asked a general question about their overall perceptions of online teaching (Table 7). Of all participants, 146 (83.0%) disagreed that online learning in dentistry is better than offline learning.

The answers were compared with participants’ perceptions of IT skills, effectiveness, challenges, and advantages related to online teaching. The overall perceptions of online teaching differed by participants’ perceptions of IT skills, effectiveness, and the advantages of online teaching (*p* < 0.05). This study showed that participants who had higher perceptions of IT skills did not believe that online teaching was better than offline teaching in dentistry. However, participants believed that online teaching could be a better teaching solution than offline teaching in dentistry in terms of the effectiveness and advantages.

A correlation analysis was performed between the perceptions of IT skills, effectiveness, challenges, and advantages with the global questions. The results showed a significant correlation between the variables. A positive correlation (r = 0.283) was demonstrated between the perceptions of IT skills and the global questions. The correlation illustrated that the higher the IT skills perceptions, the more participants chose offline teaching in dentistry. However, there were negative correlations between the global questions and perceptions of effectiveness (r = −0.257), and with perceptions of advantages (r = −0.208). The study revealed that although participants had better perceptions of the effectiveness and advantages of online teaching, they still thought that online teaching was not better than offline teaching in dentistry.

The last part of the questionnaire concerned perceptions of online teaching after experiencing online teaching during the COVID-19 pandemic (Table 8). The results showed that the faculty disagreed on whether full online or full offline learning should be used in the future, but they agreed to use some form of hybrid learning. This study also demonstrated that the faculty doubted that student competency related to expected learning outcomes was better achieved using face-to-face teaching.

## 4. Discussion

This study focused on lecturers’ perceptions from several dental schools in Southeast Asia regarding the implementation of online learning during the COVID-19 pandemic. The perceptions of online learning, in terms of IT skills and preparation, effectiveness, challenges, and advantages, were compared with participant characteristics. This study was the first to explore the topic in the Southeast Asian region. The study found that the faculty had good IT skills and perceptions of the effectiveness and advantages of online learning. However, after experiencing online learning implementation in their dental schools during the pandemic era, dental faculty members believed that online teaching was not as effective as offline teaching in the context of dental education. This study suggested that the faculty had better perceptions of the advantages and opportunities of online teaching, and recognized that the effectiveness of offline teaching alone was limited. This study also found that the faculty thought that student competency related to the expected learning outcomes would not be achieved better by using only face-to-face teaching.

Online learning utilizes information and communication technologies (ICTs) and has been implemented as a method of dental education for more than 30 years [17]. Although online courses were not popular in Asia before the pandemic, this study identified that the faculty of dental schools in the Southeast Asian region had experience in online teaching in their schools before the pandemic, and the schools had provided them with training for online teaching. The technology incorporated into teaching enhanced the learning process and was not limited by time and place [18]. The technology requires equipment to support its operation, which is not cheap; thus, acquiring technology remains a challenge for schools with different conditions of social economy [19].

The faculty agreed that more preparation was needed when performing online teaching. The sudden change to online learning during the pandemic caused dental schools which had been shut down to switch to live online classes, then pre-recorded classes [9,12]. Pre-recorded classes required instructors to spend more time preparing the learning material. However, the faculty agreed that online platforms had the tools to facilitate online classes. They also concurred that utilizing the camera when conducting online classes was important for maximizing interaction and class effectiveness. Student and teacher interactions are regarded as a key design component in online and distance learning [20]. Some studies illustrated that online learning methods were applicable for theoretical lessons and that for clinical students, it would have limitations for learning the practical or psychomotor aspect when in the laboratory or clinical setting. Furthermore, some faculty members had to maintain students’ self-regulatory behaviors, as well as e-learning motivation in conducting online classes [21].

In this study, most outcomes focused on a neutral value for the perception of the efficacy of online instruction in dentistry. Regarding online instruction for practical classes and improving student performance, faculty members were at odds. Although clinical learning can also be shifted to virtual learning by using mannequins and virtual reality/augmented reality (VR/AR)-based simulation devices to help gain clinical skills with unlimited reproducibility, there are many challenges for its implementation [22,23,24]. Since 2003, the Academic Centre for Dentistry Amsterdam (ACTA) started the development of VR and force feedback (FF)/haptic for dentistry with several lead researchers [25]. Developing abilities for some irreversible clinical procedures was the system’s goal. The system may simulate real-life situations that cause users to experience various levels of immersion and involvement, stimulating students’ senses of visual displays, aural, and force feedback [24]. The development of the system has been shown to offer training that allows users to completely concentrate on the tasks at hand, without the distraction of technical problems [24]. However, there were limitations to the system’s use for implementation at all dental schools worldwide, including terms of the curriculum, length of study, and system availability. Dental schools must update their hardware and/or software regularly to maintain the implementation process. To date, the results demonstrated that the USA and China exhibited the most publications related to the use of technology [22]. Implementing this new technology and keeping up with its rapid changes would be very challenging for dental schools in Southeast Asia.

The faculty in this study also concurred that maintaining engagement, resolving technological issues, accommodating students with hearing impairments, time management, and data protection and security are challenges of online learning. Despite these difficulties, the faculty acknowledged the benefits of online learning in terms of time and communication flexibility, low cost, and the chance to attain new skills and tools. Previous studies have suggested that the atmosphere created by online learning should be narrowly targeted, promote in-depth and meaningful teaching and learning, and include students in a more active and collaborative educational experience [1]. With all the factors related to the effectiveness, challenges, and advantages of online learning, teachers, and students needed to undergo a significant upskilling process that would be innovative and collaborative [26].

This study found that older faculty members with longer teaching experiences had lower perceptions regarding the IT skills required for online teaching. Previous studies showed that, compared to individuals from Generation Y, faculty members and students from the Baby Boomer generation and Generation X reported feeling less at ease and more anxious when utilizing technology [27]. Regarding the effectiveness and advantages of online learning, the faculty believed that it could be a better teaching solution than offline learning in dentistry. The faculty in this study agreed on the benefits of using a hybrid or blended learning approach for future teaching. This perception matches with the characteristics of current students who have effective skills and are intuitive in operating modern technology and devices [10,28]. In the era before COVID-19, the use of blended learning was limited in many dental schools worldwide, including Southeast Asia [10]. Compared to the traditional teaching approach, the implementation of a blended learning method could take more work since it requires the right attitude, a large budget, and good learning motivation from both teachers and students [12]. Improving the computer skills of both students and faculty members at each dental school should also be emphasized [12]. Communication skills that may be compromised during online learning should also be focused on when designing hybrid classes [29]. The skills of the faculty in charge of hybrid learning are also significant. More training is needed to improve students’ current educational skills and prepare in advance if similar conditions occur [12,28]. Integrating face-to-face and online learning should create a focused environment that supports deep and meaningful teaching and learning, and engages learners in a more active and collaborative educational experience [1]. The change in the learning method after the COVID-19 pandemic should have been able to maintain the quality of dental students’ education.

This study included one dental school from Indonesia, Thailand, Malaysia, and Vietnam as a representative sample of Southeast Asian countries. This study was limited by the inclusion of only one dental school from each country. The findings are based on the faculty members’ self-reported perceptions, which can be associated with a self-reporting bias based on their feelings when they completed the questionnaires. Furthermore, the answers may be influenced by the circumstances in each country regarding the pandemic. The inclusion of more dental schools to participate in the study would provide more information about faculty perceptions in the Southeast Asian region. However, the study received good responses from the faculty members of the dental schools that participated in the study, and the results based on the dental schools from each country were similar. The differences were mainly based on the age, teaching experience, and academic levels of the participants.

## 5. Conclusions

Within the limitations of this study, the Southeast Asian dental school faculty members’ perceptions were positively inclined toward integrated learning in dentistry, providing opportunities to use online learning beyond COVID-19 in the future. The discussion and research on the implementation of integrated learning in dental schools in Southeast Asian countries are important for further exploration and discussion. Schools within the region may have similar circumstances from which they can learn when designing a hybrid method; however, they must consider some changes in both technology utilization and pedagogy aspects of the method.

## Figures and Tables

**Table 1:** Study participant characteristics.

Characteristic		N	%
Gender	Male	49	27.8
	Female	127	72.2
Academic level	Lecturer	93	52.5
	Assistant Professor	33	18.8
	Associate Professor	30	17.0
	Professor	20	11.4
	Other	0	0
Experience in online teaching before the COVID-19 pandemic	Yes	84	47.7
	No	92	52.3
Training for online teaching provided by the university			
Before the pandemic	Yes	76	43.2
	No	100	56.8
During the pandemic	Yes	100	56.8
	No	76	43.2
Training for online teaching by an external institution			
Before the pandemic	Yes	32	18.2
	No	144	81.8
During the pandemic	Yes	48	27.3
	No	128	72.7
Study program responsibility (more than one answer)	Pre-clinical year	147	83.5
	Clinical year	136	77.3
	Residency	110	62.5
	Master	77	43.8
	PhD	54	30.7
Online platform for teaching being used (more than one answer)	Microsoft Teams	106	60.2
	Zoom	162	92.0
	Webex	21	11.9
	Google Classroom	113	64.2
	Others	11	7.3
Communication platform with students during online classes (more than one answer)	WhatsApp	112	63.6
	Telegram	1	0.5
	LINE	20	11.4
	Chat and call options provided bye the online platform	115	65.3
	Others	23	13.1
Communication platform with students outside online classes (more than one answer)	WhatsApp	124	70.5
	Telegram	2	1.1
	LINE	24	13.6
	Email	105	59.7
	Others	27	15.3

**Table 2:** Faculty members’ perceptions of the IT skills and preparation needed for online learning.

No.	Statement	Choice	N	%	Mean	SD	
1.	I have the IT competency to conduct online classes	Strongly disagree	0	0	4.0	0.75	Agree
		Disagree	4	2.3			
		Neutral	37	21.0			
		Agree	90	51.1			
		Strongly agree	45	25.6			
2.	Conducting online classes requires more effort in comparison to face-to-face instructions	Strongly disagree	1	0.6	3.8	0.91	Agree
		Disagree	15	8.5			
		Neutral	44	25.0			
		Agree	77	43.8			
		Strongly agree	39	22.2			
3.	Tutors have to open their cameras to maximize their live interactions with students	Strongly disagree	0	0	4.3	0.77	Agree
		Disagree	3	1.7			
		Neutral	24	13.6			
		Agree	61	34.7			
		Strongly Agree	88	50.0			
4.	Online platforms have tools to facilitate online classes	Strongly disagree	0	0	4.1	0.73	Agree
		Disagree	4	2.3			
		Neutral	26	14.8			
		Agree	92	52.3			
		Strongly agree	54	30.7			
5.	Traditional classes are more effective than online classes	Strongly disagree	2	1.1	3.7	1.00	Agree
		Disagree	16	9.1			
		Neutral	58	33.0			
		Agree	52	29.5			
		Strongly agree	48	27.3			
Total					4.0	0.46	

**Table 3:** Faculty perception of the effectiveness of online learning.

No.	Statement	Choice	N	%	Mean	SD	
1.	Theoretical classes could be taught without real interaction between tutors and their students	Strongly disagree	10	5.7	3.2	1.07	Neither
		Disagree	41	23.3			
		Neutral	41	23.3			
		Agree	70	39.8			
		Strongly agree	14	8.0			
2.	Practical classes could be taught without real interaction between tutors and their students	Strongly disagree	66	37.5	2.1	1.12	Disagree
		Disagree	60	34.1			
		Neutral	27	15.3			
		Agree	16	9.1			
		Strongly agree	7	4.0			
3.	Lack of interactions between students and their instructors results in low performance	Strongly disagree	2	1.1	4.0	0.94	Agree
		Disagree	14	8.0			
		Neutral	25	14.2			
		Agree	79	44.9			
		Strongly agree	56	31.8			
4.	Students have the facility to ask questions clearly during online lectures	Strongly disagree	1	0.6	4.0	0.84	Agree
		Disagree	6	3.4			
		Neutral	39	22.2			
		Agree	79	44.9			
		Strongly agree	51	29.0			
5.	Online classes help tutors achieve the learning outcomes of the course’s syllabi	Strongly disagree	2	1.1	3.6	0.83	Agree
		Disagree	12	6.8			
		Neutral	66	37.5			
		Agree	75	42.6			
		Strongly agree	21	11.9			
6.	Students with online learning courses outperform students with face-to-face learning	Strongly disagree	11	6.3	2.9	0.89	Disagree
		Disagree	45	25.6			
		Neutral	84	47.7			
		Agree	30	17.0			
		Strongly agree	6	3.4			
7.	Student’s participation in online courses reflects their knowledge and performance	Strongly disagree	4	2.3	3.4	0.87	Neither
		Disagree	23	13.1			
		Neutral	62	35.2			
		Agree	77	43.8			
		Strongly agree	10	5.7			
8.	Lecturers motivate students to do their assignments, and provide feedback on their assignments	Strongly disagree	0	0	4.1	0.63	Agree
		Disagree	2	1.1			
		Neutral	21	11.9			
		Agree	112	63.6			
		Strongly agree	41	23.3			
9.	Lecturers can assess students fairly and know the individual differences between them	Strongly disagree	5	2.8	3.5	0.83	Neither
		Disagree	11	6.3			
		Neutral	61	34.7			
		Agree	87	49.4			
		Strongly agree	12	6.8			
Total					3.4	0.43	Neither

**Table 4:** Faculty perceptions of the challenges of online learning.

No.	Statement	Choice	N	%	Mean	SD	
1.	Adaptability struggle	Strongly disagree	2	1.1	3.6	0.88	Agree
		Disagree	18	10.2			
		Neutral	52	29.5			
		Agree	82	46.6			
		Strongly agree	22	12.5			
2.	Technical issues	Strongly disagree	1	0.6	3.7	0.90	Agree
		Disagree	19	10.8			
		Neutral	40	22.7			
		Agree	85	48.3			
		Strongly agree	31	17.6			
3.	Internet issues	Strongly disagree	3	1.7	4.0	0.96	Agree
		Disagree	13	7.4			
		Neutral	28	15.9			
		Agree	78	44.3			
		Strongly agree	54	30.7			
4.	Organizations of work processes and time management	Strongly disagree	5	2.8	3.6	0.93	Agree
		Disagree	17	9.7			
		Neutral	38	21.6			
		Agree	92	52.3			
		Strongly agree	24	13.6			
5.	Lack of interaction	Strongly disagree	0	0	4.1	0.84	Agree
		Disagree	12	6.8			
		Neutral	20	11.4			
		Agree	87	49.4			
		Strongly agree	57	32.4			
6.	Insufficient tools for student assessment	Strongly disagree	6	3.4	3.3	1.0	Neither
		Disagree	34	19.3			
		Neutral	50	28.4			
		Agree	70	39.8			
		Strongly agree	16	9.1			
7.	Adjusting of online courses to students with hearing impairment or other disabilities	Strongly disagree	4	2.3	3.7	0.92	Agree
		Disagree	10	5.7			
		Neutral	61	34.7			
		Agree	69	39.2			
		Strongly agree	32	18.2			
8.	Data privacy and security	Strongly disagree	5	2.8	3.6	0.95	Agree
		Disagree	14	8.0			
		Neutral	57	32.4			
		Agree	72	40.9			
		Strongly agree	32	15.9			
Total					3.7	0.57	Agree

**Table 5:** Faculty perceptions of the advantages of online learning.

No.	Statement	Choice	N	%	Mean	SD	
1.	Comfort	Strongly disagree	2	1.1	3.9	0.84	Agree
		Disagree	6	3.4			
		Neutral	41	23.3			
		Agree	84	47.7			
		Strongly agree	43	24.4			
2.	Using new online learning tools	Strongly disagree	1	0.6	4.0	0.74	Agree
		Disagree	3	1.7			
		Neutral	31	17.6			
		Agree	98	55.7			
		Strongly Agree	43	24.4			
3.	E-learning is useful for grasping new skills	Strongly disagree	3	1.7	3.8	0.89	Agree
		Disagree	7	4.0			
		Neutral	53	29.0			
		Agree	76	43.2			
		Strongly agree	39	22.2			
4.	Time flexibility and communication	Strongly disagree	0	0	4.2	0.75	Agree
		Disagree	3	1.7			
		Neutral	24	13.6			
		Agree	77	43.8			
		Strongly agree	72	40.9			
5.	Low cost	Strongly disagree	2	1.1	4.0	1.01	Agree
		Disagree	14	8.0			
		Neutral	37	21.0			
		Agree	55	31.3			
		Strongly agree	68	38.6			
Total					4.0	0.60	Agree

**Table 6:** The correlation between faculty characteristics and their perceptions of online learning.

Characteristic	P1			P2			P3			P4		
	Mean (SD)	*p*	(r)	Mean (SD)	*p*	(r)	Mean (SD)	*p*	(r)	Mean (SD)	*p*	(r)
Age ^a^	46.5 (11.10)	0.00 *	−0.207	46.5 (11.10)	0.13	0.113	46.5 (11.10)	0.69	−0.030	46.5 (11.10)	0.344	−0.072
Teaching Experience ^a^	17.3 (12.16)	0.00 *	−0.230	17.3 (12.16)	0.08	0.129	17.3 (12.16)	0.85	−0.014	17.3 (12.16)	0.459	−0.056
Academic Level ^b^		0.20	−0.078		0.20	−0.069		0.80	0.015		0.882	−0.009
Lecturer	4.0 (0.43)			3.4 (0.37)			3.7 (0.59)			4.0 (0.60)		
Assist. Prof.	4.0 (0.39)			3.8 (0.41)			4.0 (0.44)			4.1 (0.69)		
Assoc. Prof.	4.1 (0.35)			3.1 (0.44)			3.4 (0.61)			4.0 (0.52)		
Professor	3.6 (0.59)			3.3 (0.40)			3.7 (0.51)			3.8 (0.57)		

Correlation analysis of the Spearman test ^a^ and Kendall test ^b^ were used, * with a significance level of *p* < 0.05. P1 Perceptions of the attitude of IT skills and class preparations of online teaching. P2 Perceptions of the effectiveness of online teaching. P3 Perceptions of the challenges of online teaching P4 Perceptions of the advantages of online teaching.

**Table 7:** The correlation between faculty members’ opinions of online learning and their perceptions of online learning.

Option	Global Question (After Experiencing Teaching during the COVID-19 Pandemic, Online Learning Is Better than Offline Learning in Dentistry)							
	P1 ^a^		P2 ^b^		P3 ^a^		P4 ^a^	
	Mean (SD)	*p*	Mean (SD)	*p*	Mean (SD)	*p*	Mean (SD)	*p*
1. Yes (n = 30)	3.7 (0.55)	0.000	3.6 (0.33)	0.001	3.5 (0.56)	0.056	4.3 (0.63)	0.006
2. No (n = 146)	4.1 (0.42)		3.4 (0.43)		3.7 (0.57)		3.9 (0.58)	

Comparative statistical analyses using the Mann–Whitney test ^a^. Independent *t*-tests ^b^ were used, with a significance level of *p* < 0.05. P1 Perceptions of the attitude of IT skills and class preparations of online teaching. P2 Perceptions of the effectiveness of online teaching. P3 Perceptions of the challenges of online teaching. P4 Perceptions of the advantages of online teaching.

**Table 8:** The faculty’s overall perceptions of online learning.

No.	Statement	Choice	N	%	Mean	SD	
1.	Student competency related to the expected learning outcome is achieved better using offline (face-to-face) teaching and learning	Strongly disagree	3	1.7	3.5	0.99	Neither
		Disagree	27	15.3			
		Neutral	53	30.1			
		Agree	66	37.5			
		Strongly agree	27	15.3			
2.	After experiencing teaching during the COVID-19 pandemic, full online teaching was chosen	Strongly disagree	31	17.6	2.5	1.04	Disagree
		Disagree	60	34.1			
		Neutral	54	30.7			
		Agree	26	14.8			
		Strongly agree	5	2.8			
3.	After experiencing teaching during the COVID-19 pandemic, full offline (face-to-face) teaching was chosen	Strongly disagree	21	11.9	2.8	1.15	Disagree
		Disagree	57	32.4			
		Neutral	48	27.3			
		Agree	34	19.3			
		Strongly agree	16	9.1			
4.	After experiencing teaching during the COVID-19 pandemic, hybrid teaching was chosen	Strongly disagree	1	0.6	4.2	0.79	Agree
		Disagree	4	2.3			
		Neutral	25	14.2			
		Agree	83	47.2			
		Strongly agree	63	35.8			

## Data Availability

The datasets used and analyzed during this current study are not publicly available due to a subsequent undergoing study but are available from the corresponding author upon reasonable request.
